# Prepregnancy Polycystic Ovary Syndrome as a Risk Factor of Subsequent Preterm Labor: A National Population-Based Cohort Study

**DOI:** 10.3390/ijerph19095470

**Published:** 2022-04-30

**Authors:** Mei-Lien Pan, Li-Ru Chen, Kuo-Hu Chen

**Affiliations:** 1 Information Technology Service Center, National YangMing ChiaoTung University, Taipei 112, Taiwan; mlpan66@nycu.edu.tw; 2 Department of Physical Medicine and Rehabilitation, Mackay Memorial Hospital, Taipei 104, Taiwan; gracealex168@gmail.com; 3 Department of Mechanical Engineering, National YangMing ChiaoTung University, Hsinchu 300, Taiwan; 4 Department of Obstetrics and Gynecology, Taipei Tzu-Chi Hospital, Buddhist Tzu-Chi Medical Foundation, Taipei 231, Taiwan; 5 School of Medicine, Tzu-Chi University, Hualien 970, Taiwan

**Keywords:** polycystic ovary syndrome, preterm labor, metformin

## Abstract

Background: Preterm labor and the following preterm births, which account for most of the perinatal deaths, are an important issue in public health. The study aims to assess the risk of subsequent preterm labor in pregnant females who have prepregnancy polycystic ovary syndrome (PCOS). Methods: This study has enrolled 1,000,000 randomly sampled females retrieved from the Taiwan National Health Insurance Research Database (NHIRD) during 1998–2012. The study excluded prepregnancy PCOS females who were initially diagnosed at age <15 or >45, and those who had inconsistent diagnoses. Moreover, the medical records of blood hormone tests, gynecologic ultrasonography, pelvic examinations, and tocometers were verified to confirm the accuracy of both diagnoses of PCOS and preterm labor. Among the prepregnancy PCOS females who became pregnant (the case group), each was age-matched to four females without prepregnancy PCOS (the control group). Results: Pregnant females in the case group (*n* = 1959) had a higher incidence of preterm labor than those in the control group (*n* = 7836) (42.98% vs. 21.99%, *p* < 0.0001). Analyzed by using logistic regression, the risk of preterm labor was significantly higher in the case group compared with the control group (crude OR: 2.674; 95% CI: 2.410–2.968, *p* < 0.0001). After adjustment with covariates, further analysis revealed a similar trend (adjusted OR: 2.405; 95% CI: 2.158–2.680, *p* < 0.0001). Among 1959 PCOS females in the case group, 196 had undergone metformin treatment. Compared with females without metformin treatment (the non-metformin subgroup), the metformin users (metformin subgroup) presented a reduced risk for preterm labor (adjusted OR: 2.238; 95% CI: 1.657–3.023). The risk of subsequent preterm labor was reduced by about 10% for the metformin subgroup compared with the non-metformin subgroup. Conclusions: Prepregnancy PCOS is an independent and significant risk factor of subsequent preterm labor. Among prepregnancy PCOS females, the risk of preterm labor is lowered by about 10% in metformin users compared with non-metformin females.

## 1. Introduction

**Figure 1:**
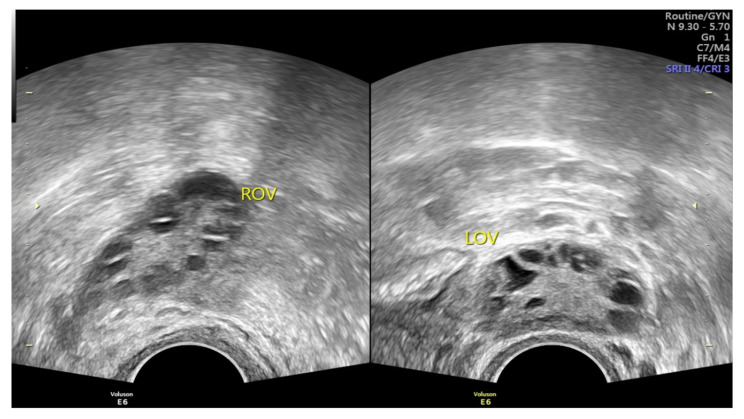
Images of PCOS under pelvic ultrasound, showing multiple immature follicles of necklace shape and/or increased volume in bilateral ovaries (Image source: Taipei Tzu-Chi hospital, Taipei, Taiwan).

Polycystic ovary syndrome (PCOS) is the most common endocrine disorder among fertile females [1,2,3], affecting approximately 4 to 14% of reproductive-age females [1,2,3,4]. As a heterogeneous disorder of chronic anovulation, it is characterized by clinical or biochemical hyperandrogenism, ovarian dysfunction, and polycystic ovarian morphology (PCOM) according to the Rotterdam criteria [2,3,4,5,6,7,8]. Under pelvic ultrasound, PCOS is defined as the findings of multiple immature follicles and/or increased volume in bilateral ovaries [4,5] (Figure 1). More than just a reproductive syndrome, PCOS is currently viewed as a disorder with metabolic consequences which could affect women’s health during different stages of life [8]. This complicated reproductive and metabolic syndrome includes insulin resistance, menstrual irregularity, infertility, androgen excess, obesity, and a chronic low-grade inflammatory state [1,7]. It is also associated with other metabolic syndromes including dyslipidemia and increases the long-term risk of type 2 diabetes mellitus [2,9,10]. Insulin resistance is prevalent among PCOS patients, and as such the probability of type 2 diabetes is 5 to 8-fold compared with females without PCOS [10,11]. Type 2 diabetes and insulin resistance are often intertwined with PCOS; however, both of these co-morbidities seem not inevitable, and can be improved under good dietary or medical control.

Although the etiologies of PCOS remain unclear and are thought to be multiple factors, the existing evidence suggests gene-associated insulin resistance is an essential cause of PCOS, with resultant hyperinsulinemia to produce excessive ovarian androgen and block follicle maturation [4,12,13,14,15]. Moreover, immunological dysfunctions, including raised levels of cytokines [12,16], autoimmune antibodies [17,18,19,20], and immune pathologies [17,21], are widespread findings among PCOS females. Currently, PCOS has been considered a state of chronic and low-grade inflammation combined with increased production of specific cytokines and chemokines such as tumor necrosis factor (TNF)α, interleukin (IL)-1, IL-6, adhesion molecules implicated in endothelial dysfunction, follistatin, and c-reactive protein (CRP) [1].

Preterm labor is usually defined as regular uterine contractions accompanied by concomitant cervical change during 20–37 weeks’ gestation [22,23,24]. Spontaneous preterm labor leads to 70% of preterm delivery [25], which is associated with 5 to 18% of pregnancies and is the leading cause of infant morbidity and mortality [23,24,25]. In the United States, approximately 12% of all live births occur before term [22,24], and preterm labor precedes approximately 50% of these preterm births, the commonest reason for hospitalization during pregnancy [22,23]. According to the WHO report, 15 million babies are born too soon every year, and more than 1 in 10 babies are born preterm, affecting families all around the world [25,26]. Accounting for most of the perinatal deaths, preterm labor, and the following preterm births place serious healthy and economic burdens on families, and are an important issue in public health [27].

In Taiwan, the prevalences of polycystic ovary syndrome and preterm labor are estimated to be around 9 and 8.5%, respectively. Clinically, the diagnosis of preterm labor is made based on regular uterine contractions noted on uterine tocometers, accompanied by changes in cervical dilation, and effacement during pelvic examinations [22]. Multiple known risk factors for preterm labor include urogenital tract infections, multiple gestation, a shortened cervix, a history of cervical conization or preterm delivery, and maternal smoking [1,22,23,24]. The pathogenesis of preterm labor is not well understood, but preterm labor might represent early idiopathic activation of the normal labor process or the results of pathological insults [24]. Theories proposed to explain the initiation of term labor include progesterone withdrawal, oxytocin initiation, and decidual activation [24]. The current understanding of this process implies that the switch of the myometrium from a quiescent to a contractile state is accompanied by a shift in signaling from anti inflammatory to pro-inflammatory pathways, which involve chemokine IL-8, cytokines IL-1 and IL-6, and contraction-associated proteins such as oxytocin receptor, connexin, and prostaglandin receptors [25]. Multiple pathologic mechanisms including intrauterine infections or external inflammation, uteroplacental ischemia, uterine overdistension or stress, and other immunologically mediated processes, can provoke decidual activation [24]. Increased expression and activity of another inflammatory cytokine TNFα, proteases matrix metal loprotease 8 (MMP-8), and MMP-9 may play a role in the process [25].

Although the past studies have reported the effects of preceding PCOS on pregnancy complications including preeclampsia and gestational diabetes, there were few studies exploring the relationship between prepregnancy PCOS and subsequent preterm labor. Due to the similar pre-inflammatory changes noted in both PCOS and preterm labor, there may be a potential connection between the two disorders. Nevertheless, many of the past studies were established on physician-identified diagnostic criteria set for PCOS and preterm labor. Thus, subjective judgment of PCOS and preterm labor by the physicians may result in selection biases when compared with objective measurement employed for the diagnoses. Moreover, many of the prior studies were performed in one health institute with either a special population (e.g., infertile females under artificial reproductive technology treatment) or a small sample, and therefore their inferences were somewhat limited and less powerful. Based on these limitations in previous studies, the current study aims to assess the risk of subsequent preterm labor among conceived females who have prepregnancy PCOS, by setting stricter diagnostic criteria to analyze a large national population-based sample.

## 2. Materials and Methods

### 2.1. The Research Ethics

As the research data solicited from the Taiwan National Health Insurance Research Database (NHIRD) has been anonymized and de-linked, relevant informed consents for the present study are waived in accordance with the local regulations. The study had been approved by the Institutional Review Board of the local hospital (Taipei TzuChi General Hospital, Taipei, Taiwan; IRB No: 10-W-069).

### 2.2. The Source of Samples and Database in the Research

Operating since 1995, the Taiwan National Health Insurance System (NHIS) has covered nearly 99% of Taiwan nationals in 2010. For diagnoses and treatments of diseases, the 9th Revision Clinical Modification of International Classification of Diseases (ICD-9-CM) and the Taiwan NHIS procedure and surgery codes are, respectively, used in the Taiwan NHI system. The analytic data in the present study were obtained from the Taiwan NHIRD, which was a national population-based research database containing inpatient and outpatient data of insured nationals, such as individuals’ demographics, dates of clinical service, the records of medical histories, medical diagnoses, medications, procedures, and surgeries. To retain the privacy of insured nationals, existent data retrieved from the Taiwan NHIRD had been de-linked by means of obliterating the identity messages of insured nationals and healthcare providers. Belonging to a special subset of the Taiwan NHIRD, the Longitudinal Health Insurance Database-2010 (LHID-2010) consisted of a longitudinal data of 1,000,000 randomly sampled nationals who had been insured in 2010, and it was specially selected for cohort studies. It had been confirmed to be not statistically different from the Taiwan NHIRD in the aspects of age, sex, and healthcare expenditures of the insured nationals.

### 2.3. Inclusion and Exclusion Criteria for Case Selection

The present study enrolled females by analyzing the de-linked and anonymous database rather than recruited participants after obtaining their informed consent. Consequently, no females would “agree” or “decline” to participate in the present study. Statistically, the main reasons for data censoring were deaths of insured nationals and their withdrawal from national insurance. After excluding those who had missing or censored data, females aged 15–45 with prepregnancy diagnoses of PCOS (ICD-9-CM code 256.4) between 1998 and 2012 were selected from the LHID-2010 subset as the case group.

Because our research focused on the risk of subsequent preterm labor (ICD-9-CM code 644.X) in females of reproductive age, those who had been diagnosed with prepregnancy PCOS at ages <15 and >45 were excluded. Established on the diagnostic criteria of PCOS mentioned above, medical records of blood hormone tests including testosterone, LH or FSH (Taiwan NHI laboratory test codes: 09064B2, 09121B, 09121C, 09078B2, 09126B, 09126C, 09078B1, 09125B, 09125C) and gynecologic ultrasonography (Taiwan NHI examination code: 19003C) were verified to confirm the diagnosis. Without the reports of gynecologic ultrasonography and blood hormone tests, the diagnoses of PCOS were not recognized as valid. Besides, females with inconsistent diagnoses of PCOS were also excluded.

**Figure 2:**
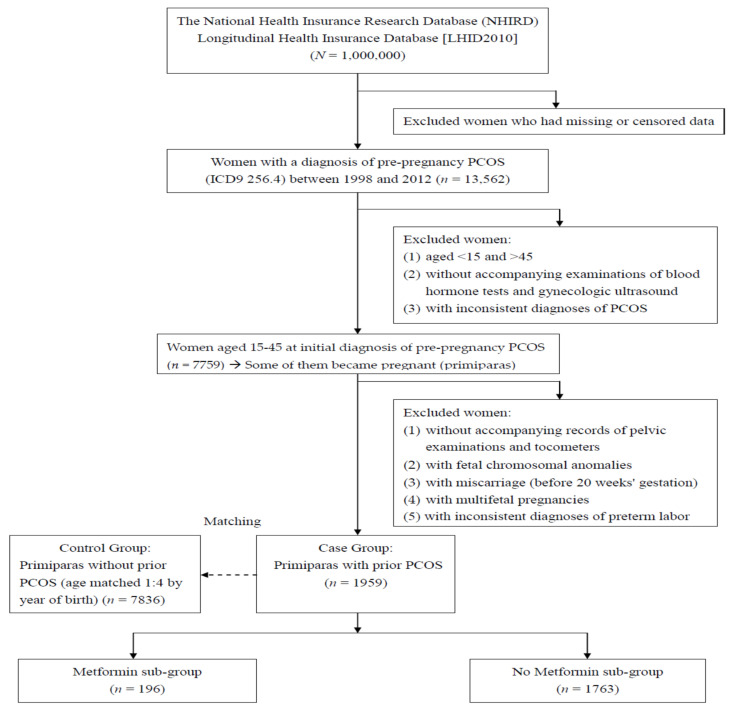
A flow chart of exclusion, inclusion, and grouping in the females with and without prepregnancy PCOS.

In the second stage, we analyzed the prepregnancy PCOS females who became pregnant (primiparas) and had preterm labor after 20 weeks’ gestation. Females with fetal chromosomal anomalies or inconsistent diagnoses of preterm labor, and those without accompanying records of pelvic examinations (Taiwan NHI examination code: 55021C) and tocometers (Taiwan NHI examination code: 18013, 18014, 18035) were excluded. Fetal chromosomal anomaly is a major confounding factor for preterm labor, and thus affected pregnancies were not included in the current study to prevent interference. Females with multifetal pregnancies (ICD-9-CM code 651.X), a well-known risk factor of preterm labor were also excluded in this stage. Moreover, the diagnosis of preterm labor was recognized as invalid without accompanying records of pelvic examinations [23] and tocometers. Based on reports of blood hormone tests, ultrasonic findings, pelvic examinations and tocometers, the ICD-9-CM coding, as well as diagnoses of PCOS and preterm labor, are much more strict and precise. In the third stage, every pregnant female with prepregnancy PCOS (Case Group) was matched by age to 4 females without prepregnancy PCOS (Control Group), as shown in Figure 2, a flowchart of exclusion, inclusion, and matching of the females with and without prior PCOS.

The current study also investigated the effects of metformin usage in the prepregnancy PCOS group on the risk of subsequent preterm labor. By acting as an insulin sensitizer, metformin can lower blood sugar and androgen levels, thus, improving hyperglycemia, hyperandrogenism to restore ovulation and treat PCOS [15,28]. The prepregnancy PCOS females were then divided into two subgroups according to metformin use or not (Figure 2).

For women in Case and Control Groups, their general characteristics including ages at the first pregnancy and the PCOS diagnosis, occupations, urbanization, economic status, and co-morbidities were surveyed. “Age at the first pregnancy” represents the mean years of the females when they initially conceived. “Age at the PCOS diagnosis” represents the mean years of the females when PCOS had been initially diagnosed. The demographics “degree of urbanization” was categorized as urban, suburban, and rural. “Occupation” was classified into white and blue collar, retired, or others. According to their levels of insurable wages under the Taiwan NHIS, the economic status of nationals could be classified into four levels: insurable wage ≥NTD 40,000; NTD 20,000–40,000; <NTD 20,000; retired/others (exchange rate of the currency: 27.91 NTD = 1 USD). Being potential influential factors, co-morbidities could be considered as below: hypertension (ICD-9-CM code: 401–405.X); diabetes mellitus (ICD-9-CM code: 250.X); dyslipidemia (ICD-9-CM code: 272.X); cerebrovascular diseases (ICD-9-CM code: 430–438.X); chronic pulmonary diseases (ICD-9-CM code: 490–496.X); ischemic heart diseases (ICD-9-CM code: 410–414.X); and autoimmune diseases. Autoimmune diseases include SLE (ICD-9-CM code: 710.0), Sjogren’s syndrome (ICD-9-CM code: 710.2), rheumatoid arthritis (ICD-9-CM code: 714.0; 714.30–714.33), systemic sclerosis (ICD-9-CM code: 710.1), autoimmune *thyroiditis* (ICD-9-CM code: 245.2), Behcet’s diseases (ICD-9-CM code: 136.1), vasculitis (ICD-9-CM code: 446.X; 443.1), Kawasaki diseases (ICD-9-CM code: 446.1), pemphigus (ICD-9-CM code: 694.4), dermatomyositis (ICD-9-CM code: 710.3), polymyositis (ICD-9-CM code: 710.4), Crohn’s diseases (ICD-9-CM code: 555.X) and ulcerative colitis (ICD-9-CM code: 556.0–556.6; 556.8–556.9).

### 2.4. Data Analyses

In the current study, SAS^®^ (Cary, NC, USA) software version 9.4 was used for analyses of our research data. Pearson χ^2^ and Student t test were employed to compare the general characteristics (age, demographics, and co-morbidities) of females between the case and control groups, as appropriate. Furthermore, logistic regression analysis was performed to calculate the odds ratios (ORs) and 95% conference intervals (CIs) for the risks of preterm labor after being adjusted with potential influential factors. A level of *p* value < 0.05 was considered significant.

## 3. Results

The characteristics of the enrolled females in the case and control groups are shown in Table 1. The mean years at the PCOS diagnosis was 27.51 in both groups. Females in the Case Group had an older mean age at first pregnancy than those in the Control Group (30.25% vs. 27.41%, *p* < 0.0001). For the characteristics economic status, urbanization, and occupation, the dominant populations in both groups were insurable wage 20,000–40,000 NTD (>40%), urban (>60%), and white collar (>55%), respectively. Pregnant females between Case and Control Groups had significant differences in their economic status (*p* < 0.0001), occupation (*p* < 0.0001), urbanization (*p* = 0.0008). In the characteristics co-morbidities, females in Case Group had a higher incidence of hypertension (*p* = 0.0415), dyslipidemia (*p* < 0.0001) and diabetes mellitus (*p* < 0.0001) compared with those in Control Group (Table 1).

Table 2 presents an analysis of the risk of subsequent preterm labor in females with or without prepregnancy PCOS. Pregnant females in the Case Group had an elevated incidence of preterm labor in comparison to those in the Control Group (42.98% vs. 21.99%, *p* < 0.0001). Analyzed by using logistic regression, the risk of preterm labor was higher in the Case Group compared to the Control Group (crude OR: 2.674; 95% CI: 2.410–2.968, *p* < 0.0001). After adjustment with covariates, further analysis revealed a similar trend (adjusted OR: 2.405; 95% CI: 2.158–2.680, *p* < 0.0001). The covariates used for adjustment in the logistic regression analyses included age at the first pregnancy, urbanization, economic status, occupation, and co-morbidities (diabetes mellitus, hypertension, and dyslipidemia). They were chosen as adjustment covariates based on the statistical significance of the comparison between the case and control groups. Except the item PCOS (OR: 2.674; 95% CI [2.410–2.968]), each aforementioned covariate had no significant effect on the research focus (preterm labor) (all *p* > 0.05). Similarly, except the items metformin use (OR: 2.499; 95% CI [1.871–3.337]) and non-metformin use (OR: 2.695; 95% CI [2.418–3.003]), each aforementioned covariate had no significant effect on the research focus (preterm labor) (all *p* > 0.05).

In the PCOS (*n* = 1959) and control (*n* = 7836) groups, there were 78 and 90 nationals among whom gestational diabetes were diagnosed. Among 196 metformin users in the PCOS (case) group, 89 had the diagnosis of GDM. In contrast, among 1763 non-metformin users in the PCOS (case) group, 25 had the diagnosis of GDM.

Table 2 also shows the subgroup analysis for prepregnancy PCOS women in the Case Group. In a total of 1959 cases, 196 women had undergone metformin treatment (the metformin subgroup). In comparison to those without metformin treatment (the non metformin subgroup), the metformin users (metformin subgroup) presented a reduced risk of preterm labor (adjusted OR: 2.238; 95% CI: 1.657–3.023). The risk of subsequent preterm labor was lowered by about 10% for the metformin subgroup in comparison to the no metformin subgroup.

## 4. Discussion

The results of the national population-based study demonstrate that prepregnancy PCOS females have a higher incidence of preterm labor compared with females without previous PCOS (42.98% vs. 21.99%, *p* < 0.0001). Further analysis reveals that females with previous PCOS have a >2-fold elevated risk of preterm labor than those without previous PCOS (adjusted OR: 2.405; 95% CI: 2.158–2.680). Other covariates, including age at the first pregnancy, urbanization, economic status, occupation, and co-morbidities (diabetes mellitus, hypertension, and dyslipidemia) had no significant effect on preterm labor (all *p* > 0.05). The prepregnancy PCOS females are at an increased risk of preterm labor in their subsequent pregnancies, and therefore they should be provided applicable information and suggestion to facilitate early management and specialty referral. For females at risk, consultation and measures should be arranged in responding to the possible physiological effect and psychological impact. During pregnancies, enhanced antepartum surveillance, as well as facilitated access to higher levels of neonatal institutes, can be considered for prepregnancy PCOS females.

The conclusions of the previous studies which investigated the relationship between PCOS and preterm labor are conflicting. Our results are similar to those which deem PCOS as a risk factor for preterm labor [1,2,8]. The current understanding of term or preterm labor implies that the underlying mechanisms involve intra-amniotic infection, decidual senescence, and breakdown of maternal-fetal tolerance, in all of which elevated cytokines and chemokines play a key role [1,25]. As mentioned above, PCOS *per se* has been viewed as a state of chronic low-grade inflammation with increased production of specific cytokines and chemokines such as TNFα [16,29], IL-1, IL-6, follistatin, CRP, and adhesion molecules involved in endothelial dysfunction [1]. Thus, these cytokines and chemokines may mediate further actions occurring in preterm labor and may be an important linkage between these two pathologies. Moreover, decreased progesterone levels [30] and NK cell dysfunction [31], which are relatively common in patients with PCOS, can accelerate the decidual senescence and breakdown of maternal-fetal tolerance to induce preterm labor. Consequently, the up-regulated apoptosis and gradual tissue destruction might end in the clinical manifestations of the disease. Currently, biochemical and molecular findings have supported the theory that labor may be regarded as part of an inflammatory cascade model, which involves pathways of specific leukocytes and proinflammatory mediators (cytokines and chemokines) [1]. The common pathways will be activated physiologically in case of term labor, whereas some diseases such as infection and PCOS can activate one or more of the components of the common pathways in the case of preterm labor [25]. Although the causes of preterm labor are multiple factorial and can’t be explained in a simple way only, the hypothesis mentioned above may provide reasonable explanations for the results observed in the present study. Nonetheless, the infrastructural mechanisms of preterm labor seem complicated and remain to be clarified.

Insulin is not used in sole PCOS without the complication of diabetes mellitus. For PCOS with insulin resistance, metformin can be used to improve insulin sensitivity by increasing the activity of insulin receptor tyrosine kinase, which further activates post-receptor insulin signaling pathways at a molecular level. In the literature, there exist oppositional results with regard to the effects of metformin on preterm labor [2,27,32,33,34]. Our results reveal that among prepregnancy PCOS females the risk of preterm labor is reduced by about 10% in metformin users compared with non-metformin females. The females with PCOS are featured by hyperandrogenism, hyperinsulinemia, low endometrial glycodelin levels, and resulting hypofibrinolysis via elevated plasminogen activator inhibitor (PAI) activity [35,36]. A recent investigation has shown significantly lower serum glycodelin and insulin growth factor binding protein 1 (IGFBP-1) levels in the pregnancies of PCOS women, suggestive of a deficient endometrial environment for the maintenance of pregnancy [36]. As a biguanide drug, metformin can ameliorate chronic anovulation and reactive hyperinsulinemia in PCOS patients [15,28,37] by improving insulin sensitivity [32]. It is postulated that the positive effect of metformin on preterm labor originates from increasing endometrial glycodelin, a kind of glycoprotein functioning as tissue adhesion, implantation, and continuance [35]. Likewise, previous research has revealed that metformin ameliorates many key markers regarding endometrial receptivity and blood supply among PCOS women [36]. Another possible mechanism for metformin to reduce spontaneous preterm labor could involve the mammalian target of rapamycin complex 1 (mTORC1) signaling pathway, which has a key role in the timing of birth, as its activation induces preterm birth, and its inhibition prevents or postpones the onset of labor. Metformin inhibits mTORC1 by AMP activated protein kinase [33]. Metformin may be helpful in coping with several risk factors of preterm labor [35]. However, many unknown aspects in the subtle actions of metformin warrant further investigation.

The strengths of this national population-based study lie in a relatively large size of the sample, a considerate method of sampling, and rigorous inclusion/exclusion criteria in females with a diagnosis of PCOS or preterm labor. In contrast to prior research, an important point of the present study is our sample, which has been retrieved from a nationwide population database of a broad survey instead of purposive samples noted in several prior studies. Thus, our study results are more robust due to a large sample size and a reduction of possible sampling bias in the research process. Moreover, both diagnoses of PCOS and preterm labor in all enrolled females are established according to the objective records of blood tests, gynecologic ultrasonography [5], pelvic examinations, and uterine tocometers in contrast to the individual and subjective judgment of the physicians. As much as possible, the overall biases resulting from these human factors and research processes are minimalized.

The present study has some inherent limitations, because there is lacking in several demographic datasets within the nationwide database, such as body mass index, marital and social status, cigarette or alcohol consumption, and usage of self-paid drugs, we are unable to investigate the influence of the aforementioned factors. Secondly, using an ICD-9-CM code cannot entirely reflect the real clinical condition after all. Based on the uncertainty of defining a disease from administrative data, blood tests, gynecologic ultrasonography, pelvic examinations, and uterine tocometers have been verified to confirm the diagnosis. Thirdly, in the Taiwan NHI system medical and administrative data are declared mainly for reimbursement. Therefore, the inconsistency in the sequential data may affect the research results. Moreover, relevant information of metformin dosage and duration is unavailable in the Taiwan NHIRD, which is an innate and unavoidable limitation of the national database. Finally, the current study cannot inspect the effect of races on preterm labor due to the ethnic similarity in Taiwan.

## 5. Conclusions

The results of this national population-based study demonstrate that prepregnancy PCOS is a significant and independent risk factor for subsequent preterm labor. Furthermore, among prepregnancy PCOS females, the risk of preterm labor is reduced by about 10% in metformin users compared with non-metformin females. Relevant information and suggestion should be provided to affected females to facilitate early management and specialty referral. For females at risk, consultation and measures can be considered while responding to the potential physiological effect and psychological impact. During pregnancies, enhanced antepartum surveillance, as well as facilitated access to higher levels of neonatal institutes, can be considered for prepregnancy PCOS females.

## Figures and Tables

**Table 1:** Characteristics of pregnant females with and without prepregnancy PCOS.

	Case Group		Control Group		Statistics	
	Women with Previous PCOS		Women without Previous PCOS			
	(*n* = 1959)		(*n* = 7836)			
	*n*	%	*n*	%	OR [95% CI]	*p*-Value
**Age at PCOS diagnosis (y/o)**	27.51 ± 4.84		27.51 ± 4.84			1.0000
15–25	672	34.30	2688	34.30		
26–35	1188	60.64	4752	60.64		
36–45	99	5.05	396	5.05		
**Age at first pregnancy (y/o)**	30.25 ± 4.42		27.41 ± 4.54			0.0001 ***
**Occupations**						<0.0001 ***
White collar	1233	62.43	4391	56.06		
Blue collar	326	16.64	1405	17.93		
Retired or others	410	20.93	2040	26.03		
**Urbanization**						0.0008 **
Urban	1323	67.53	4951	63.185		
Suburban	511	26.08	2375	30.31		
Rural	125	6.38	510	6.51		
**Economic status (insurable wages)**						<0.0001 ***
≥40,000 NTD	443	22.61	1259	16.07		
20,000–40,000 NTD	838	42.78	3292	42.01		
<20,000 NTD	406	20.72	1954	24.94		
Retired and others	272	13.88	1331	16.99		
**Co-morbidities**						
Diabetes mellitus	87	4.44	103	1.31	3.49 [2.61–4.66]	<0.0001 ***
Hypertension	35	1.79	94	1.20	1.50 [1.01–2.22]	0.0415 *
Dyslipidemia	95	4.85	221	2.82	1.76 [1.37–2.25]	<0.0001 ***
Ischemic heart disease	27	1.38	97	1.24	1.12 [0.73–1.71]	0.6191
Cerebrovascular disease	14	0.71	97	1.24	0.57 [0.33–1.01]	0.0504
Chronic pulmonary disease	307	15.67	1097	14.00	1.14 [0.99–1.31]	0.0589
Autoimmune disease	136	6.94	457	5.83	1.20 [0.99–1.47]	0.0653

* *p* < 0.05, ** *p* < 0.001,*** *p* < 0.0001, by chi-square test or student *t* test, as appropriate. Data are expressed as the number (%) or mean ± standard deviation, as appropriate.

**Table 2:** Analysis of the risk of subsequent preterm labor in females with/without prepregnancy PCOS.

	No preterm Labor		Preterm Labor		Statistics	
	*n*	%	*n*	%	Crude OR ^a^	Adjusted OR ^b^
					[95% CI]	[95% CI]
**Group**						
Control Group: (*n* = 7836)	6113	78.01	1723	21.99	Reference	Reference
women without PCOS	1117	57.02	842	42.98	2.674 * [2.410–2.968]	2.405 * [2.158–2.680]
women with PCOS						
*Subgroup in Case Group*						
*Non Metformin Subgroup*	1002	56.83	761	43.17	2.695 * [2.418–3.003]	2.409 * [2.154–2.695]
(*n* = 1763)						
*Me* *tformin Subg* *roup*	115	58.67	81	41.33	2.499 * [1.871–3.337]	2.238 * [1.657–3.023]
(*n* = 1763)						

^a^ Odds ratio (OR) and 95% confidence interval [95% CI] are calculated by logistic regression analysis, as compared to the reference group. ^b^ Adjusted for the covariates: age at the first pregnancy, urbanization, economic status, occupation and co-morbidities. * *p* < 0.0001.

## Data Availability

Regarding data availability, the data utilized in this study cannot be made available in the manuscript, the supplemental files, or in a public repository due to the Taiwan Personal Information Protection Act, but the data from the National Health Insurance Research Database is available for application from the National Health Research Institutes in Taiwan for researchers who meet the criteria for data access. Further information regarding data access is available at the National Health Research Institutes website (http://nhird.nhri.org.tw, accessed on 5 January 2022) or by email to nhird@nhri.org.tw.
